# Effects of a Mobile App to Promote Social Participation on Older Adults: Randomized Controlled Trial

**DOI:** 10.2196/64196

**Published:** 2024-09-30

**Authors:** Kenjiro Kawaguchi, Atsushi Nakagomi, Kazushige Ide, Katsunori Kondo

**Affiliations:** 1 Department of Social Preventive Medical Sciences Center for Preventive Medical Sciences Chiba University Chiba Japan; 2 Research Department Institute for Health Economics and Policy Tokyo Japan

**Keywords:** gerontology, geriatrics, older adults, elderly, older people, community dwelling older adult, aging, social participation, walking, mHealth, apps, smartphone, digital health, digital technology, digital interventions, physical activity, exercise

## Abstract

**Background:**

Social participation is crucial for healthy aging, improving physical and mental health, cognitive function, and quality of life among older adults. However, social participation tends to decline with age due to factors like loss of social networks and health issues. Mobile health apps show promise in promoting healthy behaviors among older adults, but their effectiveness in increasing social participation remains understudied.

**Objective:**

This randomized controlled trial aimed to evaluate the efficacy of a mobile app called Encouragement of Social Participation (ESP, “Shakai Sanka no Susume;” Hitachi) in promoting social participation and physical activity among community-dwelling older adults.

**Methods:**

The study recruited 181 community-dwelling adults aged 60 years or older from 2 municipalities in Japan and through a web-based research panel. Participants were randomly assigned to either the intervention group (n=87), which used the ESP app for 12 weeks, or the control group (n=94), which used only Google Fit. The ESP app incorporated features such as self-monitoring of social participation, personalized feedback, gamification elements, and educational content. Primary outcomes were changes in social participation frequency over the previous 2 months and changes in step counts, measured at baseline and week 12. Secondary outcomes included changes in specific types of social activities and subjective well-being. Data were analyzed using analysis of covariance and linear mixed-effects models.

**Results:**

The intervention group showed a significantly greater increase in social participation frequency compared with the control group (adjusted difference 3.03; 95% CI 0.17-5.90; *P*=.04). Specifically, the intervention group demonstrated higher frequencies of participation in hobbies (adjusted difference: 0.82; 95% CI 0.01-1.63) and cultural clubs (adjusted difference 0.65; 95% CI 0.07-1.23) compared with the control group. However, there were no significant differences in weekly step counts between the groups. Subgroup analyses suggested potentially larger effects among participants who were older than 70 years, female, had lower educational attainment, and were recruited from community settings, although only females and the lower educational attainment subgroups demonstrated 95% CIs that did not encompass zero.

**Conclusions:**

The ESP mobile app effectively promoted social participation among community-dwelling older adults, particularly in hobbies and cultural club activities. However, it did not significantly impact physical activity levels as measured by step counts. These findings suggest that mobile apps can be valuable tools for encouraging social engagement in older populations, potentially contributing to healthy aging. Future research should focus on optimizing app features to maintain long-term engagement and exploring strategies to enhance physical activity alongside social participation.

**Trial Registration:**

University Medical Information Network Clinical Trial Registry UMIN000049045; https://center6.umin.ac.jp/cgi-open-bin/ctr_e/ctr_view.cgi?recptno=R000055781

## Introduction

Social participation, involvement in activities that facilitate interactions with others in the community [1], is a key determinant of healthy aging [2]. Social participation benefits older adults, improving their physical activities [3,4], physical and mental health [5-8], cognitive function [9,10], and overall quality of life [11]. Despite these well-established benefits, social participation declines with age owing to factors such as the loss of social networks [12] and health issues [13]. Therefore, interventions that can effectively promote and maintain social participation among older adults are needed.

There has been a growing interest in the potential of digital technologies, particularly mobile apps, to promote healthy aging [14,15]. A survey conducted by the Ministry of Internal Affairs and Communications of Japan revealed that more than 60% of Japanese adults aged 70 years and older own smartphones [16], indicating a rapidly rising trend in smartphone ownership among older adults in Japan. Research suggests that mobile health interventions can effectively promote physical activities [17,18], self-management of chronic conditions [19,20], and well-being [21] in this demographic. These findings suggest that smartphones and apps are accessible and scalable interventions that can reach a broader population of older adults and enhance their health and well-being.

The development of apps that integrate evidence-based strategies for behavior change is one promising approach to increasing social participation among older adults [22]. An observational study showed that a mobile app designed to encourage social participation among community-dwelling older adults was acceptable to them, with approximately 60% expressing a willingness to continue using the app [23]. A quasi-experimental study [24] examined the effects of a mobile app on the stimulation of social participation among older adults, showing that it increased the intention for social participation and frequency of social participation among community-dwelling older adults. According to a systematic review [25], incorporating features such as performance feedback and encouragement, self-monitoring capabilities, and educational content may enhance the effectiveness of health behavior promotion apps for older adults. The incorporation of behavior change techniques, such as self-monitoring and social comparison, also improves engagement and outcomes [26,27]. By designing apps that incorporate these techniques, we can create apps that facilitate more successful behavior change.

Despite the potential of apps to encourage social participation and promote healthy aging, limited research examines the effects of mobile health interventions on social participation [24]. This study introduces a novel app that integrates evidence-based features specifically designed to promote behavior change among older adults. In contrast to earlier apps [22-24], our app integrates evidence-based features, such as self-monitoring of social participation, personalized feedback and encouragement, gamification elements, and educational content regarding the benefits of social participation, all based on established behavior change theories. The primary objective of this study was to evaluate the efficacy of the app in promoting social participation and physical activity (as measured by step count) among community-dwelling older adults. We hypothesized that the use of the app would lead to a significant increase in the frequency of social participation compared with the control group. The secondary objective was to assess the effects of the app on specific types of social activity and subjective well-being.

## Methods

### Study Design

We conducted a randomized controlled trial (allocation ratio of 1:1) to determine the efficacy of an app that promoted social participation among community-dwelling older adults. In the intervention group, we provided participants with an investigational app for 12 weeks.

### Participants

The participants were community-dwelling older adults from 2 different sources. First, we invited older adults residing in 2 municipalities to participate in this study. The first municipality is a small town with a population of 10,000, while the second is a city with a population of about 500,000 in two prefectures near Tokyo. We distributed information about the study through posters, flyers, and municipal public relations magazines with the cooperation of the staff at the municipal office’s Department of Senior Services. We held briefing sessions for those interested in participating and explained the research content in detail. After explaining the research objectives comprehensively, we selected participants who met the inclusion criteria and expressed a willingness to participate. Second, we recruited a web-based research panel from the Internet survey research company ASMARQ Co, Ltd. The registrants for the web-based research panel who met the inclusion criteria received an email invitation to participate in this study. The company continued sending email invitations until 100 participants were obtained.

The inclusion criteria were as follows: (1) community-dwelling older adults aged 60 years or older, who (2) used a smartphone daily, (3) spoke Japanese, (4) usually used LINE (LY Corporation; one of the most popular apps for social communication in Japan [16]), and (5) were not certified as requiring support or nursing care under long-term care insurance in Japan. We excluded participants who used a senior smartphone (featuring simplified menus, enlarged text, and icons) from this study because of the inappropriate functioning of the social participation app.

### Procedure

**Figure 1:**
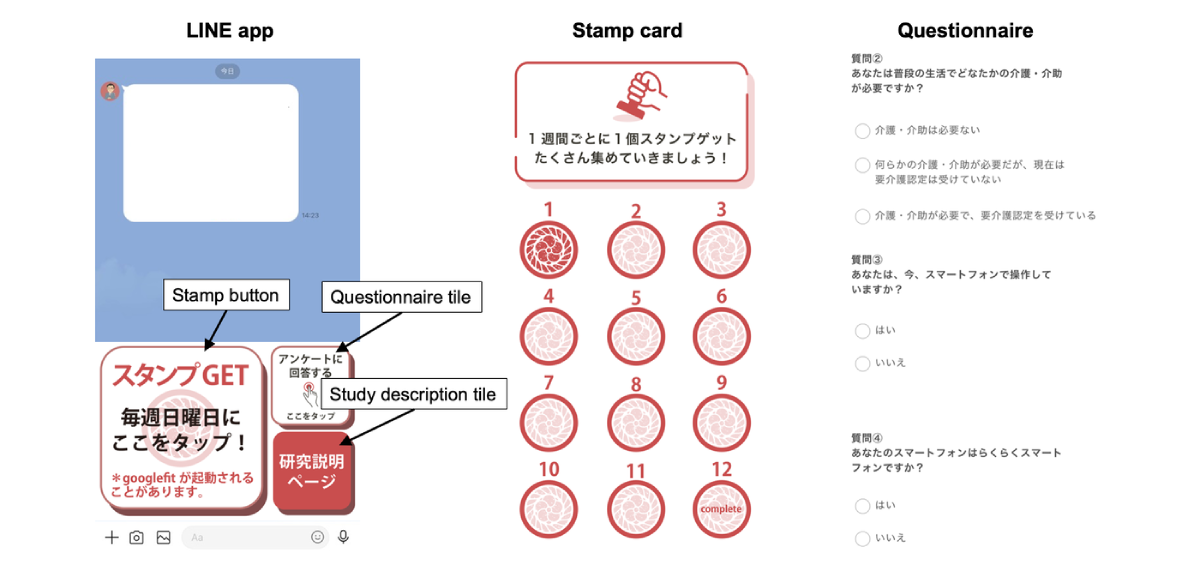
Example of the LINE app interface customized for this study, including the main screen, stamp card, and questionnaire.

We used the messenger app LINE to communicate with the participants in the intervention and control groups. In Japan, more than 90% of individuals use LINE as their primary social media platform [16]. Users can create their accounts using their phone numbers or email addresses and connect with friends through LINE ID. The participants were asked to connect their LINE ID with our research account. We sent a welcome message and research description to the participants’ accounts and obtained their informed consent through checkboxes on the app. The participants could send messages to our research team through LINE to ask questions or withdraw their consent. To maintain their motivation and commitment to our study, we implemented a stamp card system to track their progress (Figure 1). The participants earned 1 stamp per week, for a total of 12 stamps over 12 weeks. We sent weekly reminder messages to prompt participants to press the stamp tile and use the app.

We instructed the participants in the intervention group to install and use the Encouragement of Social Participation (ESP) app on a weekly basis, in addition to installing Google Fit to obtain step count data. By contrast, the control group only installed Google Fit and occasionally used it. We asked the participants to fill in the app-based questionnaires at the beginning and end of the study to obtain baseline information and assess outcomes. We sent invitations to complete the questionnaires to the participants in both groups through LINE at the beginning and after 85 days of the study.

### Randomization

After providing informed consent, participants were promptly assigned to either the intervention or control group based on a random number generated by a computer-based randomization system at the backend of the LINE app. The study investigators were blinded to the group allocation. This random allocation process ensured that the group assignments were not predictable in real time.

### Intervention: Encouragement of a Social Participation Smartphone App

**Figure 2:**
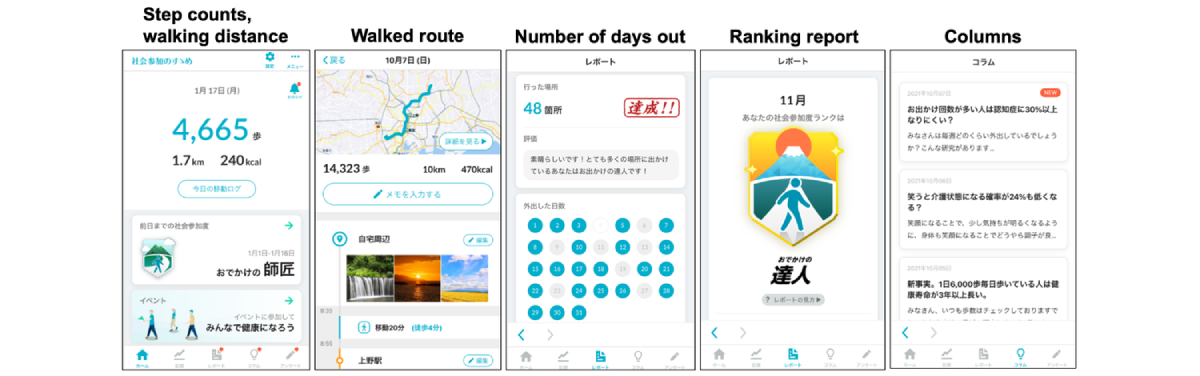
Example of the Encouragement of Social Participation smartphone app interface, including the step counts, walking distance, walked route, number of days out, ranking report, and educational columns.

The ESP app (“Shakai Sanka no Susume”) was developed by Hitachi. This free app is compatible with both the Android (Google) and iOS (Apple Inc) operating systems. It incorporates several key features designed to promote healthy behaviors (Figure 2). It facilitates self-monitoring of social participation through features such as feedback and encouragement (based on social participation levels) and educational content that emphasizes the benefits of social participation. Furthermore, the app uses smartphone data such as location information, step counts, and other relevant data to measure outdoor activities. It tracks the user’s movements using GPS and displays the walking distance, step counts, walked route, and number of locations visited on a map along with the step counts. This enables self-monitoring of social participation and physical activity. The app uses a specific classification algorithm based on the user’s average step count, number of places visited, and number of days spent outside. This algorithm categorizes the user’s social participation level into 4 categories, that are “Expert,” “Master,” “Skilled,” and “Beginner.” This gamification element aims to increase user motivation and engagement. Furthermore, the ESP app delivers weekly digestible columns to users. These columns inform users about the importance and value of social participation based on previous research findings and serve as an educational component to raise awareness and reinforce the importance of staying socially active. In the preliminary testing phase, the pilot app, featuring only GPS tracking and column delivery, was evaluated among a small group of older adults in a rural area, indicating an increase in the distance traveled. We were not involved in the development of this app.

### Control: Google Fit App

The control group used the Google Fit app, a widely available free health and fitness tracking app developed by Google for Android and iOS. The app allows users to monitor their physical activities, such as walking, running, and cycling and track various metrics, such as distance, time, and calories burned. We instructed the participants in the control group to occasionally use Google Fit to track their physical activity levels and monitor their progress. We did not inform the control participants of the details of the ESP app, including the product name.

### Outcome Measures

The study’s primary outcomes were (1) changes in social participation frequency over the previous 2 months and (2) changes in step counts, both measured at baseline and week 12. Participants in both groups completed app-based baseline and follow-up questionnaires to assess their frequency of social participation. The survey asked, “Over the last two months, how many times did you participate in the following groups or activities?” The participants reported their participation frequency in 6 types of group activities, such as volunteering, sports, hobbies, cultural clubs, salons, and others. We selected these activities based on the Long-term Care Needs Survey template provided by the Ministry of Health, Labor and Welfare. We summed the frequency in the 6 activities for each participant, and the difference during the 12 weeks represented the change in the frequency of social participation over the previous 2 months.

In addition, we used the participants’ step counts as another primary outcome to evaluate the effect of the ESP app on walking. Step counts were measured using Google Fit for both the intervention and control groups, regardless of the smartphone operating system. We calculated the weekly average of daily step counts for each participant by dividing the total number of steps recorded in a given week by the number of days with valid step count data in that week as tracked by Google Fit. We excluded daily step counts with fewer than 100 steps [28].

The secondary outcomes included (1) changes in the social participation frequency for each activity (volunteering, sports, hobbies, cultural clubs, salons, and other activities) over the previous 2 months; (2) changes in the frequency of going out weekly over the previous month; and (3) changes in subjective well-being, including happiness, life satisfaction, and purpose in life (rated on a scale of 0-10, with higher scores indicating greater well-being).

In the follow-up questionnaires, we asked the participants how often they used the ESP app to measure their intensity of use.

### Sample Size

We based the sample size on a previous study [24]; we assumed that social participation was 19.5 times per 2 months in the intervention group and 15 times per 2 months in the control group, with an SD of 14 and a dropout rate of 12.5%. Therefore, we required 350 participants for a 2-tailed α level of 5% and a statistical power of 80%.

### Statistical Analysis

We described the baseline characteristics of the study participants, including sex, age, educational attainment, marriage, instrumental activities of daily living (IADLs), self-reported health, social participation frequency, frequency of going out weekly, and subjective well-being. All continuous values were expressed as mean (SD), and categorical variables were reported as numbers (percentages). We assessed IADLs using the 5-item Tokyo Metropolitan Institute of Gerontology Index of Competence [29], which includes questions on public transportation, shopping, meal preparation, bill payment, and banking (yes or no). Responses (0-5) were summed, with higher scores indicating greater independence. We considered the mean and SD for continuous variables and the number and proportion for categorical variables. We conducted all analyses on an intention-to-treat basis.

The primary analysis used analysis of covariance (ANCOVA) to compare the change in social participation frequency between the intervention and control groups, considering the effects of baseline variables, including sex, age, IADLs, and social participation frequency as covariates [30]. This study reported the adjusted mean difference in the change in social participation frequency between the 2 groups, along with a 95% CI. In addition, we compared weekly changes in step counts from weeks 1 to 12 between groups, using covariate-adjusted linear mixed models [31]. We considered the average weekly step counts to be nested within individuals with random intercepts. This approach enabled the analysis of longitudinal data with repeated measurements while accounting for the correlation between observations from the same individual. The model included fixed effects for the group (intervention or control), time (weeks), an interaction between group and time, and covariates of sex, age, and IADLs. The interaction term indicates the difference in the weekly steps between the groups in a specific week. The normal distribution of each primary outcome variable was confirmed using the Shapiro-Wilk test.

We compared the secondary outcomes between the groups using ANCOVA adjusted for baseline variables, including sex, age, IADLs, and outcomes. In addition, we conducted subgroup analyses, stratified by sex, age (dichotomized as ≤70 or >70 years based on the median age of the sample), educational attainment (below high school, high school, or above high school), and the recruitment source (community or web-based research panel), to investigate potential differences in the app’s effects among several demographic subgroups [32]. We calculated the *P* values for the interaction of the group with each baseline variable using the primary ANCOVA model.

Further, we conducted an ad hoc analysis to compare changes in weekly step counts from week 1 to each subsequent week (weeks 2-12) between groups. This analysis aimed to investigate the short-term effects of the ESP app on physical activity levels, which may not have been captured in the primary analysis. We performed an ad hoc analysis using the same linear mixed model approach as in the primary analysis.

All tests were 2-sided; we considered significance at a *P* value of less than .05. We used R version 4.2.1 (R Foundation for Statistical Computing).

### Ethical Considerations

The ethics committees of the Japan Agency for Gerontological Evaluation Study (approval number 2022-01) and Chiba University (approval number M10635) reviewed and approved the study protocol. All participants provided informed consent to participate in this study through checkboxes on LINE app. No personally identifiable information such as name or address was collected. Participants were offered an electronic gift certificate for JPY ¥1500 (US $10.38) as an honorarium.

## Results

### Participant Characteristics

**Figure 3:**
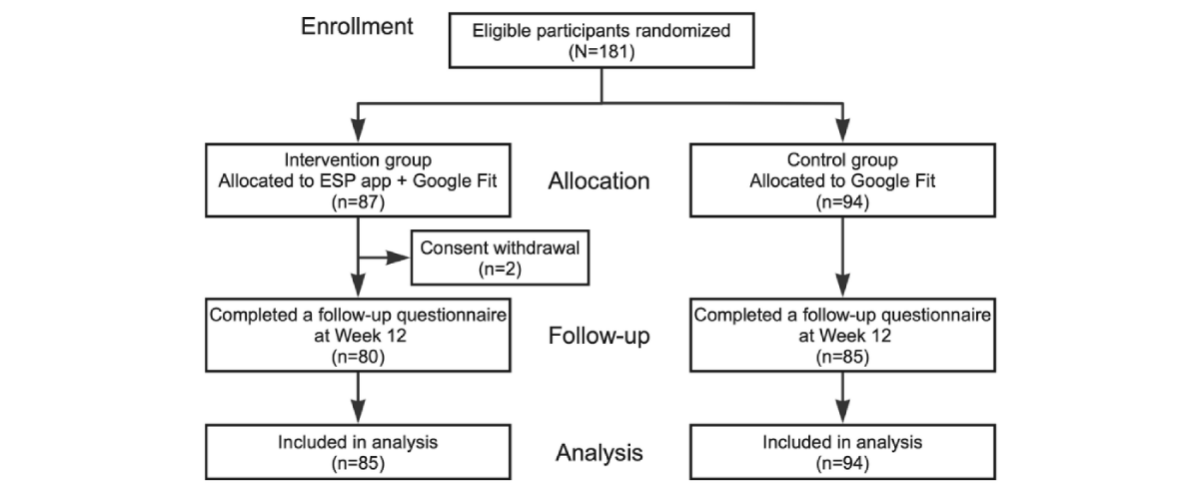
Flowchart of participant selection for analysis. ESP: Encouragement of Social Participation.

Figure 3 shows a flowchart of the participant selection between October 2022 and August 2023; 181 participants were recruited (81 from the community and 100 from internet survey registrants). In October 2023, the app’s developer introduced an update that removed the feature providing digestible columns on social participation. To preserve the consistency and integrity of the intervention, we decided not to recruit additional participants, even before reaching the preplanned sample size of 350. A post hoc power analysis revealed that our sample size provided a power of 63.5% to detect the observed effects with an α level of 0.05. Of the 181 participants, we assigned 87 and 94 to the intervention and control groups, respectively. In addition, 2 participants in the intervention group withdrew their consent because of health concerns, leaving 179. All the participants completed the baseline questionnaires, whereas 80 and 85 in the intervention and control groups, respectively, completed the follow-up questionnaires, resulting in a follow-up rate of 92.2% (165/179). The baseline characteristics of the participants in the intervention and control groups were balanced (Table 1). The mean age of the study participants was approximately 70 years, 45% (80/179) were male, 70% (126/179) were educated beyond high school, 80% (143/179) were married (their mean IADL score was 4.6), and 90% (159/179) reported good health. The mean overall frequency of participation in any form of social activity was 12.28. Sports had the highest mean frequency of participation in specific activities (3.99), followed by volunteering (2.21), hobbies (21.98), salons (1.85), other activities (1.23), and cultural clubs (1.02). The mean study period was 86.9 days. Regarding the use of the ESP app, 25% (20/80) of participants in the intervention group reported minimal or no usage, 6% (5/80) reported using it several times a month, and 69% (55/80) used it at least once per week (Table 2).

**Figure 4:**
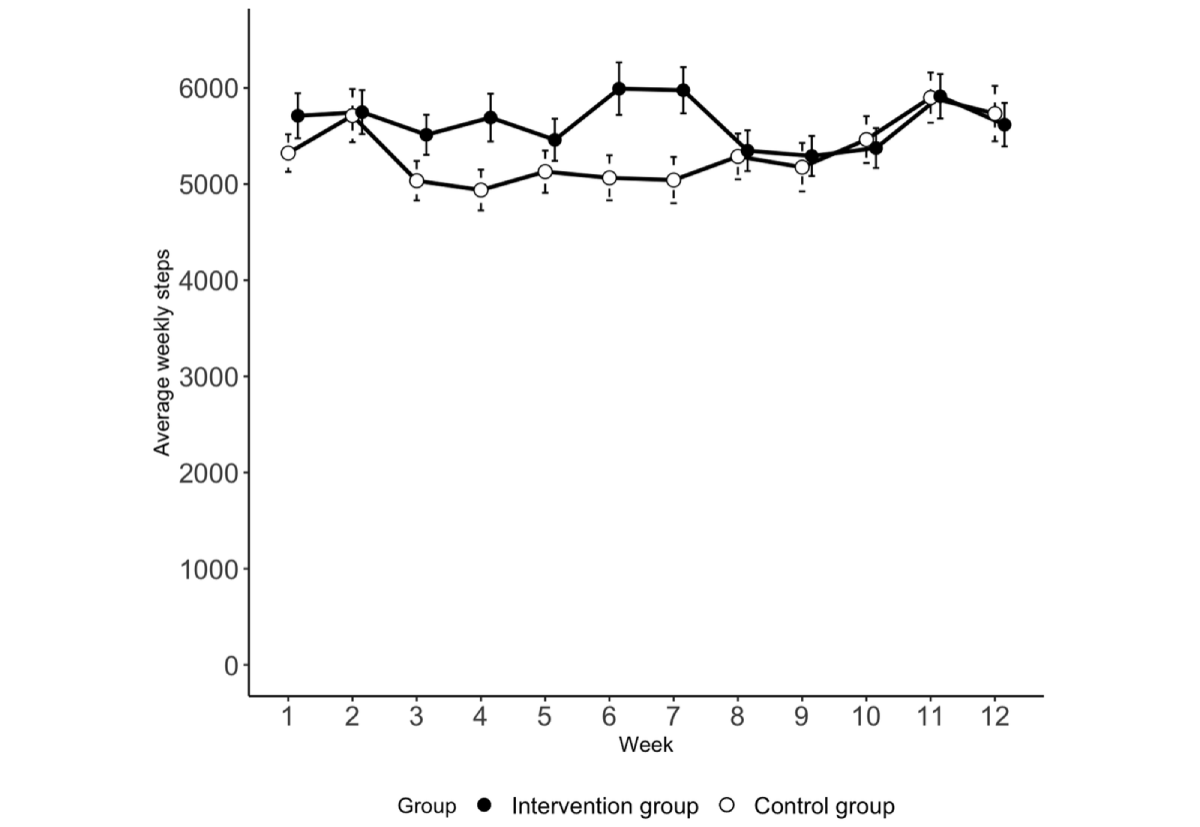
Average weekly step counts (error bars denote the SE of the mean).

Of the 179 participants, 155 (86.6%) recorded their daily step count at least once during the 12 weeks. On average, each participant recorded valid step counts (ie, daily step counts ≥100) for 40 days. The average daily step count was 5474 (SD 4743). Between weeks 3 and 7, participants in the intervention group took more steps daily than those in the control group (differences in step counts between the 2 groups ranged from 330 to 934); however, after week 8, almost no difference was observed, or slightly more steps were observed in the control group (Figure 4 and Table 3).

**Table 1:** Baseline characteristics of the study participants.

Characteristics		Total (N=179)	Intervention (n=85)	Control (n=94)
Age (years), mean (SD)		70 (6.3)	69.9 (6)	70.1 (6.7)
Male, n (%)		80 (44.7)	43 (51)	37 (39)
**Educational attainment, n (%)**				
	High school or less	53 (29.6)	20 (26)	33 (35)
	Above high school	126 (70.4)	65 (77)	61 (65)
Married, n (%)		143 (79.9)	71 (84)	72 (77)
IADL^a^ (0-5, higher scores indicating more independence), mean (SD)		4.6 (0.7)	4.7 (0.7)	4.7 (0.7)
**Self-reported health, n (%)**				
	Poor	5 (2.8)	4 (5)	1 (1)
	Fair	15 (8.4)	5 (56)	10 (11)
	Good	137 (76.5)	67 (79)	70 (74)
	Very good	22 (12.3)	9 (11)	13 (14)
**Social participation frequency over the previous 2 months, mean (SD)**				
	Any	12.28 (13.06)	11.16 (10.50)	13.30 (14.99)
	Volunteering	2.21 (4.40)	1.61 (3.19)	2.76 (5.22)
	Sports	3.99 (5.82)	4.02 (5.63)	3.96 (6.01)
	Hobbies	1.98 (2.95)	1.78 (2.71)	2.17 (3.15)
	Cultural clubs	1.02 (2.06)	0.79 (1.54)	1.23 (2.42)
	Salons	1.85 (3.49)	1.79 (2.95)	1.90 (3.93)
	Other activities	1.23 (3.06)	1.18 (3.61)	1.28 (2.49)
Frequency of going out weekly over the previous month, mean (SD)		6.41 (4.10)	6.07 (3.49)	6.72 (4.58)
**Subjective well-being, mean (SD)**				
	Happiness	7.52 (1.75)	7.54 (1.80)	7.50 (1.71)
	Life satisfaction	7.44 (1.82)	7.45 (1.80)	7.44 (1.85)
	Purpose in life	7.40 (1.88)	7.34 (1.80)	7.45 (1.95)

^a^IADL: instrumental activities of daily living.

**Table 2:** Frequency of use of the Encouragement of Social Participation app in the intervention group.

Frequency	Intervention (n=80), n (%)
Not used	8 (10)
Rarely used	12 (15)
Several times a month	5 (6)
Once a week	10 (13)
2-3 days a week	14 (18)
4 times or more a week	31 (39)

**Table 3:** Average weekly step counts.

Group	Week											
	1	2	3	4	5	6	7	8	9	10	11	12
Intervention, mean (SE)	5710 (234)	5748 (229)	5512 (209)	5692 (249)	5460 (219)	5992 (272)	5976 (241)	5347 (212)	5292 (209)	5375 (207)	5913 (231)	5618 (226)
Control, mean (SE)	5322 (195)	5712 (276)	5034 (205)	4938 (212)	5129 (219)	5065 (234)	5042 (241)	5287 (238)	5175 (252)	5463 (243)	5900 (262)	5733 (288)
Group × Week^a^	—^b^	–565 (.15)	63 (.87)	74 (.85)	–333 (.41)	245 (.54)	396 (.33)	–220 (.59)	–208 (.61)	–330 (.42)	–266 (.52)	–340 (.42)

^a^Data are presented as values of interaction terms between groups and weeks (*P* value).

^b^Not applicable.

### Primary Outcomes

#### Social Participation

The frequency of social participation over the 2 months changed by +1.69 in the intervention group and –2.45 in the control group. The intervention group had a significantly greater increase in social participation frequency compared with the control group, with an adjusted difference of 3.03 (95% CI 0.17-5.90, *P*=.04).

#### Step Counts

The results of the linear mixed-effects model demonstrated that the interaction between the group and week 12 was insignificant (*P*=.53), indicating no significant difference in weekly steps between the groups.

### Secondary Outcomes

Table 4 presents the secondary outcomes. The intervention group showed higher mean changes compared with the control group for several activities, with notable differences observed for hobbies and cultural clubs (95% CIs not including zero). For other activities, including volunteering, sports, salons, and going out, the intervention group showed trends toward increased participation, but the 95% CIs included zero. Regarding subjective well-being measures (happiness, life satisfaction, and purpose in life), the intervention group showed small increases compared to the control group, though the 95% CIs for these measures also included zero.

**Table 4:** Effects of promotion of social participation by the Encouragement of Social Participation app.

Outcome		Mean changes during 12 weeks		
		Intervention	Controls	Adjusted difference^a^ (95% CI)
**Social participation**				
	Volunteering	0.15	–0.66	0.29 (–0.51 to 1.09)
	Sports	–0.18	–1.11	0.89 (–0.43 to 2.22)
	Hobbies	0.75	–0.26	0.82 (0.01 to 1.63)
	Cultural clubs	0.68	–0.28	0.65 (0.07 to 1.23)
	Salons	0.05	0.00	0.20 (–0.80 to 1.20)
	Other activities	0.24	–0.14	0.36 (–0.41 to 1.13)
	Going out	0.71	–0.65	1.11 (–0.03 to 2.25)
**Subjective well-being**				
	Happiness	0.04	0.00	0.09 (–0.28 to 0.46)
	Life satisfaction	–0.01	–0.08	0.12 (–0.30 to 0.54)
	Purpose in life	0.18	0.00	0.22 (–0.25 to 0.70)

^a^Adjusted for sex, age, instrumental activities of daily living, and baseline outcome variables using analysis of covariance.

### Subgroup Analysis

We conducted subgroup analyses to investigate whether the effects of the ESP app on promoting social participation varied across participant characteristics. Subgroups were defined based on age (≤70 and >70 years), gender, education level (high school or less and above high school), and recruitment source (community and web-based research panel).

Table 5 presents the adjusted differences and 95% CIs for each subgroup, along with *P* values for interactions. While some subgroups showed trends toward increased social participation in the intervention group, most 95% CIs included zero. Notable exceptions were the female subgroup and the high school or less education subgroup, where the 95% CIs did not include zero.

**Table 5:** Effects of the Encouragement of Social Participation app on social participation by subgroups.

Subgroup			Mean changes during 12 weeks							
			Intervention		Controls		Adjusted difference^a^ (95% CI)		*P* value for interaction	
**Age (years)**										.24
	≤70	1.42		–1.48		1.53 (–1.33 to 4.41)				
	>70	2.00		–3.59		4.68 (–0.68 to 10.04)				
**Sex**										.67
	Male	0.12		–4.00		2.34 (–2.57 to 7.25)				
	Female	3.33		–1.33		3.62 (0.15 to 7.43)				
**Educational attainment**										.38
	High school or less	2.79		–0.93		3.59 (0.39 to 6.79)				
	Above high school	–1.59		–5.38		–0.23 (–7.30 to 7.01)				
**Recruitment source**										.35
	Community	1.49		–4.29		4.50 (–0.12 to 9.13)				
	Web-based research panel	1.88		–0.65		2.01 (–1.21 to 5.24)				

^a^Adjusted for sex, age, instrumental activities in daily living, and the baseline outcome variable by an analysis of covariance.

### Ad Hoc Analysis: Short-Term Effects on Physical Activity

We conducted an ad hoc analysis to investigate the short-term effects of the ESP app on physical activity levels. We compared the weekly step counts from weeks 1 to 8 between the groups using the same linear mixed model approach as in the primary analysis, with fixed effects for group, week, and their interaction, and random intercepts for individuals. Table 3 shows that some of the interaction terms between the group and weeks (weeks 2-8) yielded positive results whereas no interaction terms did not reach statistical significance at *P* value of <.05. This indicates that the differences in step counts might not differ clearly between the intervention and control groups in any week.

## Discussion

### Principal Findings

This randomized controlled trial evaluated the efficacy of the ESP app in promoting social participation and physical activity among community-dwelling older adults. The results indicated that participants who used the ESP app during the 12-week study significantly increased the frequency of social participation compared to the control group. Furthermore, the intervention group showed higher frequencies of participation in hobbies and cultural clubs than the control group. Conversely, the intervention group did not show an increase in weekly step counts.

Several key features in the app may explain the positive effect of the ESP app on social participation. First, the app’s self-monitoring capabilities enable users to easily track and visualize their social participation levels, providing feedback that may increase their awareness and motivation to engage in more social activities. Previous interventional research reported that the goal-setting and self-monitoring features of a tablet app aimed at improving balance and strength helped motivate two-thirds of older participants [33]. The inclusion of educational content on the benefits of social participation or other health topics may also have helped to increase knowledge, motivation, and skills among this demographic, as several studies have demonstrated [34,35]. Finally, a gamification element that displays achievement levels of social participation may enhance engagement with the app.

The observed step counts were higher for ESP app users than for the control group from week 3 to 7, suggesting that interventions aimed at promoting social participation can have spillover effects on physical activity [3,36]. However, these effects did not lead to continued increases in step count over the 12-week period. These temporary effects may be attributed to the decrease in novelty of the app wearing off over time [37,38]. When participants first started using the app, the excitement of trying the new technology and the motivation to engage in social activities may have led to an initial increase in physical activity levels. However, the participants may have become less interested in exploring the app’s features or less responsive to the feedback and encouragement provided by the app as the study progressed. Furthermore, the app’s gamification elements (achievement levels and rewards) may have diminished as users became accustomed to the mechanism [39]. Although extrinsic rewards can promote engagement, relying on these alone may not be effective in the long term.

Future development of mobile health apps like the ESP app should consider a multifaceted approach to maintain long-term user engagement and promote lasting behavior changes. Research suggests that cultivating intrinsic motivation maintains a user’s interest and involvement over an extended period [40]. According to self-determination theory, intrinsic motivation can be fostered by ensuring the fulfillment of 3 basic psychological needs, that are autonomy, competence, and relatedness [41]. Autonomy, the need for self-control over behavior and goals [41], can be supported by allowing users to set personal goals and choose methods, with customizable features [42]. Competence, the need to feel effective and capable [41], can be enhanced by offering challenges suited to the user’s skill level and constructive feedback on progress, similar to gamification elements. Relatedness, the need for social connection [41], can be promoted through social features that enable progress sharing, mutual support, and community engagement [43]. Addressing these needs may foster intrinsic motivation and maintaining long-term engagement in app users, leading to more effective interventions. In addition, developing a hybrid model that combines digital tools with human interaction could enhance intervention effectiveness and user engagement, as suggested by human-computer interaction literature [44]. Furthermore, incorporating artificial intelligence–powered support features, such as chatbots or AI-assisted personal guides, could provide scalable human-like interaction, potentially increasing user engagement as shown in recent studies [45]. Combining these elements, such as intrinsic motivation cultivation, human support integration, and artificial intelligence–powered features, could promote continued engagement with mobile health apps aiming at behavior change.

We did not observe the differences in the step counts between the intervention and control groups. The study’s sample size was likely not sufficiently large to detect a significant difference in step counts between the groups [46], given the considerable variability in daily step counts among the participants (SD 4743). It is also possible that the use of Google Fit as a step counter in both the intervention and control groups contributed to the lack of significant differences. Google Fit is a widely used and well-established app that provides users with step-tracking and other physical activity-monitoring features. Its presence in the control group may have inadvertently led to an increased awareness and motivation to engage in physical activity among the control participants [47], thereby reducing the potential differences between the 2 groups.

Notably, the effect sizes were larger among participants who were 70 years or older, female, had lower educational attainment, and recruited from community settings. This suggests that these subgroups may benefit more from the ESP app. According to a previous study that examined the usage patterns of apps and wearables to support physical activity in a sample of Japanese-speaking individuals [48], the app users were more likely to be younger, male, and have a higher level of education. The participants in these subgroups in the current study may have had less previous experience with physical activity apps before joining the study. Therefore, the ESP app may have had a greater effect on social participation among the older, female, and lower educational subgroups. In addition, the larger effect sizes observed among female participants may be due to gender-based variations in preferences for app designs and features. A previous study that investigated design preferences for an electronic mental health program, aimed at preventing depression [49], revealed that women preferred user-responsive and guided formats. Conversely, men preferred video game–type designs. These findings suggest that tailoring the design, features, and content of an ESP app to the preferences and needs of specific subgroups can enhance its impact on social participation.

### Limitations

This study had several limitations. First, the ESP app was updated during this study and the company removed the feature that delivered digestible columns for social participation, which may have led to smaller effects of the app on social participation. In addition, due to the update, we halted the recruitment of study participants before we reached the planned sample size, which may have reduced the statistical power to detect significant effects. Second, the participants were selected from the local community and through a web-based research company. The use of the research panel may have excluded older adults who are less comfortable with information and communication technology or have lower digital literacy. These specific recruitment sources and methods may have resulted in a sample that was not representative of the diverse Japanese older adult population. This recruitment process may limit the generalizability of the findings to a broader older adult population. Third, this study included a 12-week follow-up period, which did not allow the assessment of long-term effects. Further research is needed to evaluate the long-term sustainability of these effects and explore strategies for optimizing engagement with apps over time. Fourth, a relatively low adherence to step-count tracking was observed. On average, each participant recorded valid step counts (ie, daily step counts ≥100) for only 40 days out of the 84-day study period. The incomplete data may have reduced the accuracy and representativeness of the step count findings. Therefore, the step count results should be interpreted with caution. Fifth, the study relied on self-reported measures of social participation, which may have been subject to recall bias. Further studies should incorporate objective measures of social activity, such as GPS tracking or check-in systems, to complement self-reported data. Finally, a quarter of the participants in the intervention group reported minimal or no usage of the app. This relatively low level of engagement may have led to an underestimation of the app’s effect. However, it may also reflect real-world conditions, where not all users fully engage with health interventions.

### Conclusion

The findings of this study suggest that the ESP app promotes social participation among community-dwelling older adults. The intervention group showed a significant increase in the self-reported frequency of social participation compared with the control group, indicating the app’s efficacy. This increase in social participation highlights the app’s potential as an affordable and expandable method for promoting social participation and healthy aging. Thus, the ESP can promote social participation among older adults and may contribute to healthy aging. Further research and modifications are warranted to optimize its effectiveness and maintain its effects.

## Appendix

Multimedia Appendix 1 CONSORT checklist.

## Abbreviations

ANCOVA: analysis of covariance
ESP: Encouragement of Social Participation
IADL: instrumental activity of daily living
